# Acetylcholinesterase, pro-inflammatory cytokines, and association of *ACHE* SNP rs 17228602 with male infertility

**DOI:** 10.1371/journal.pone.0282579

**Published:** 2023-04-07

**Authors:** Khulah Sadia, Mbah Ntepe Leonel Javeres, Faheem Tahir, Syed Tahir Abbas Shah, Rabia Habib, Zahid Muneer, Sabir Hussain, Syed Muhammad Nurulain

**Affiliations:** 1 Department of Biosciences, COMSATS University Islamabad, Islamabad, Pakistan; 2 Institute of Medical Research and Medical Plants Studies, Yaounde, Cameroon; 3 Public Health Laboratories Division, National Institute of Health (NIH), Islamabad, Pakistan; University of Hyderabad, INDIA

## Abstract

Male infertility is a complex and polygenic reproductive disease. 10–15% of the males are affected by idiopathic infertility conditions. Acetylcholine (ACh), a major neurotransmitter has been reported to play a non-neuronal role as well. Acetylcholinesterase (AChE) is the primary ACh hydrolyzing enzyme whose over or lower expression influence the availability of ACh for physiological roles. The purpose of the study was to find the possible impact and association of acetylcholinesterase, *ACHE* gene variant rs 17228602, and pro-inflammatory cytokines in clinically diagnosed infertile males. The study includes clinically diagnosed fifty non-infertile (control) and forty-five infertile males. Whole blood AChE enzymatic activity was measured. Genotyping of rs17228602 was carried out from peripheral blood by standard molecular methods. Pro-inflammatory cytokines were determined by the ELISA method. AChE enzyme was found to be significantly elevated in infertile than non-infertile males. *ACHE* SNP rs17228602 had shown significant association in dominant model (odd ratio = 0.378, 95% CI = 0.157–0.911, p-value 0.046). Pro-inflammatory cytokine IL-1β was notably increased with statistical significance (p ≤0.05) in male infertile patients. The study concludes and speculates that AChE plays role in the pathogenesis of male infertility through the modulation of inflammatory pathways. Further studies in this direction may resolve the idiopathic cases of male infertility. Other variants of *ACHE* and the association of miRNA for the regulation of AChE in male infertility are suggested for further insight.

## Introduction

Infertility is a reproductive physiological disorder characterized by the failure to get pregnant after twelve months of having regular non-fruitful sex. The male factor of infertility is about 40–50% [1]; however, the figure varies in different populations and countries. According to the World Health Organization estimate, about 190 million individuals are struggling with infertility issues and the number is progressively growing [2]. Male infertility accounts for 22% in Pakistan, out of which primary and secondary infertility is 4% and 18% respectively [2]. Globally, idiopathic and unexplained male infertility factor is about 40–70% [3], despite advancements in diagnostic areas. The routine diagnostic procedure to identify male infertility is a physical examination, medical and life history, and semen analysis. In addition, the common genetic screening involves cystic fibrosis transmembrane conductance (CFTR) gene and karyotype analysis [4]. Nonetheless, the current genetic testing and approaches are limited, even though there might be several genetic, epigenetic, and miRNA regulatory factors that are affected in infertility conditions and may function as an investigative tool for unexplained and idiopathic sterility cases.

Among the scores of physiological factors for infertility, cytokines modulations and oxidative stress have garnered attention in the recent past [5–9]. Meanwhile, an association of the cholinergic system with inflammatory pathways is well-known and documented in the literature [10–12]. The Association and correlation of cholinergic enzymes and oxidative stress have also been reported in the literature [12–15]. The cholinergic system includes brain neurotransmitter ACh and its hydrolysing enzymes; acetylcholinesterase (AChE) and butyrylcholinesterase (BChE). Although the primary physiological role of ACh and AChE is neuronal, evidence shows their presence and role in non-neuronal organs like pancreatic alpha cells [16], endothelial cells [17], placental cells [18, 19], bladder [20], lymphocytes [21, 22], spleen [23]. AChE enzyme is found in red blood cells at high concentrations. In human sexual health, ACh has been shown to present and play a role in penile erection and relaxation by stimulation of nitric oxide (NO) from endothelial cells and then inhibition of NA release [24, 25]. Along with other neurotransmitters, it is also correlated with the central control of ejaculation. AChE has been observed in spermatozoa and its presence is linked with sperm motility. Ammar *et al*., [26] reported elevated AChE activity in teratozoospermia cases of infertility and found an association with apoptosis. Cholinergic molecules are expressed in Leydig and Sertoli cells of the male reproductive system. It is reported that AChE decreases the level of testosterone in Leydig cells [27].

Several genes that are found to be related to infertility have been discovered with the development in techniques of molecular biology which may be helpful in the establishment of diagnostic tools and therapeutic methods intended for male infertility. However, the acetylcholinesterase gene (*ACHE)* variant has not been studied in this connection. *The ACHE* gene is considered conserved amongst populations. Exome Aggregation Consortium reported approximately 141 synonymous mutations and 137 missense variants for the *ACHE* gene [28]. *ACHE* encoding gene spans about 6kb and is located at chromosomal position 7q22. This gene consists of six exons and four introns. 13 SNPs of this gene have been identified in population-based studies. SNP rs17228602 is positioned on 3’UTR of *the ACHE* gene. SNPs positioned at 3’UTR can influence recognition sequences of miRNAs which are non-coding short RNA regulatory molecules. About 244 miRNAs have been identified as potential targeting different cholinergic transcripts [29]. Numerous molecular pathways are regulated by miRNA by post-transcriptional silencing of the gene. In a study, the AChE-R variant was found to be overexpressed in transgenic mice which lead to reduced sperm quantity and motility, eventually causing infertility [30]. Notably, ACh regulates inflammation in peripheral nervous systems by modulation of cytokines [31].

Cytokines are the kind of signalling molecules involved in testicular function and male reproductive health [32, 33]. Cytokines are either pro-inflammatory or anti-inflammatory, the former causing inflammation while the latter helps in reducing inflammation. Activated macrophages in the host system produce pro-inflammatory cytokines which upregulate the inflammatory reactions. The leading pro-inflammatory cytokines include IL-1β, IL-6, and TNF-α. Pro-inflammatory cytokines impart a significant role in balancing and regulating the blood-testis barrier. IL-6, TNF-α, and IL-1β affect tight junctions present in Sertoli cells [34, 35]. Escalation in pro-inflammatory cytokines is linked with an increase in seminal levels of ROS [36].

In the present study, the possible role of AChE activity and pro-inflammatory cytokines were examined in infertile males that are clinically diagnosed and investigated the tentative association of *ACHE* gene SNP rs 17228602 with male infertility. To the best of our knowledge, no study with cholinergic interplay in male infertility is reported earlier. This study will help in understanding unexplained infertility cases with new approaches, and open a new direction for further studies like miRNA regulating AChE and cytokines in infertile, ultimately may lead to the development of a novel diagnostic marker for male infertility.

## Materials and methods

### Ethical approval

Ethical approval for the study was obtained (CIIT/Bio/ERB/19/88) from the departmental ethical review board (ERB), Department of Biosciences, COMSATS University Islamabad, and written consent was taken from participants of the study. Only clinically diagnosed infertile males in the age range of 23–45 years were selected for the study. It was ensured that they were not undergoing any prescribed treatment for their infertility at the time of inclusion. Participants suffering from known infectious and chronic diseases like diabetes, neurological disorders, liver dysfunction, and cancers were excluded. Men with smoking habits and other known addictions were also excluded.

#### Study subjects and specimen collection

Fifty fertile males who have at least one child and typical semen parameters were included in the control group. Forty-five infertile volunteers were recruited from the Bioclinical Laboratory Islamabad and the National Institute of Health (NIH), Islamabad. Semen samples were collected by masturbation after 4–7 days of sexual abstinence. Semen analysis for sperm concentration and motility was done according to the World Health Organization (WHO) criteria. To perform biochemical and molecular analysis, three ml venous blood was obtained from volunteers and collected in two EDTA-k vacutainers (Atlas; Labovac Italiano, FL Medical), one for separation of plasma and the other for AChE determination and genotype analysis. Plasma was used for the pro-inflammatory cytokines determination.

Samples were kept at 4°C for further use.

### Chemicals and primer designing

Primers for the selected *AChE* SNP were constructed through Primer 3 software (version 0.4.0). NCBI Blast was applied to inspect the sensitivity of the primer pairs (https://www.ncbi.nlm.nih.gov/tools/primer-blast/). For the further testing, *in silico* PCR Tool was employed to ensure the specificity of the primer pairs designed. Specific primers were then bought from Macrogen Inc. (Rockville, MD, USA). Table 1 lists the sequences along with the product size of primers used for SNP profiling. Required chemicals for biochemical and molecular analysis were obtained from Thermo Fisher Scientific (Waltham, MA, USA) and Sigma-Aldrich (St Louis, MO, USA).

**Table 1:** The sequence of Primer for *AChE* SNP rs 17228602.

Primer sequence	Tm	Product size
F: 5’GAGGAGGAGAAAAGAATGACC3’	56.9	365bp
R: 3’TCCTCTAATGAGTGGTCGGAC5’	59.2	

### Semen analysis

Media Lab CASA (Computer Assisted Semen Analysis) software was used for semen analysis which follows WHO guidelines (2010). Semen samples were placed in a dry heat incubator for approximately 30 minutes. Samples were evaluated after tender mixing for parameters such as volume, viscosity, colour, pH, particulate debris, presence of epithelial cells, sperm motility, and sperm concentration. Semen volume was measured by graduated test tubes. The viscosity of semen was assessed by inserting a glass rod in the sample and considering the length of thread formed by removing the rod. For normal samples, the range of thread is 2 cm. Based on the semen consistency, the sample was considered to have standard or non-standard viscosity [37]. A clean Maker counting chamber was used for sperm concentration and motility analysis at room temperature. Sperm concentration was categorized according to the WHO guidelines (2010). A normal semen profile has more than 40 million sperm/ejaculate, >20 million sperm/mL, ≥50% motility, and more than 30% sperms with standard form.

### RBC-AChE determination

Acetylcholinesterase in blood was measured by Worek *et al*., 1999 [38], based on Ellman’s method [39] at 436 nm. For this purpose, blood dilutions were prepared by mixing 30ul of blood and 3ml of diluting reagent (triton-x added phosphate buffer) for each sample. All reagents; 2ml phosphate buffer (0.1M) of pH 7.4, 10 μl Ethopropazine, 100 μl DTNB of (10 mM), and 1ml of blood dilutions were mixed and then kept incubated at 37°C for 20 minutes. After incubation, 50 μl acetylthiocholine substrate was added and mixed into the cuvettes. The optical density of the reaction mixture was obtained per minute for 5 minutes using spectrophotometer (Model “Specord 50 plus” Number; 233H1280C manufactured by Analytic Jena, made in Germany).

### DNA extraction and SNP genotyping

**Fig 1:**
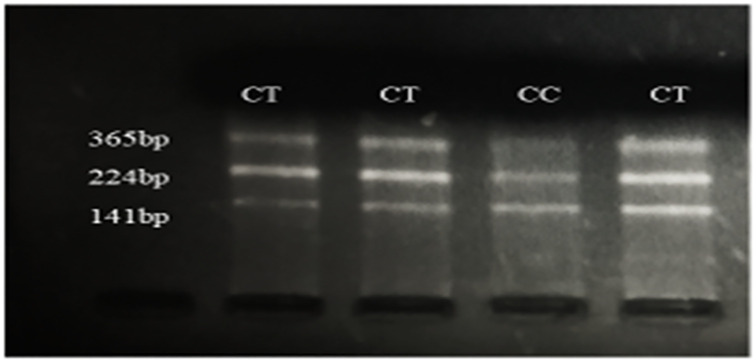
Identification of SNP rs17228602. Visualization of PCR products after digestion with restriction enzyme Psp5II (PpuMI) are shown on 2% ethidium bromide-stained agarose gel. Genotypes are shown above the lane and fragment size to the left of figure.

Lahiri and Nurnberger, 1991 [40] method was used to extract genomic DNA from whole blood. The method included cell lysis followed by digestion of protein and then precipitation of DNA. Quantification of DNA was done via nanodrop (Implen Pearl Nano-Photometer; Thomos Scientific), followed by suspension in Tris-EDTA buffer and finally kept at −20°C. Genotyping of rs17228602 was executed by PCR-RFLP followed by forward and reverse primers. Primer sequences are given in Table 1. The polymerase chain reaction was performed with an overall volume of 25μL comprising of 2ul genomic DNA, 0.5ul DNTPs, 3ul MgCl_2_, 0.5μl each of forward and reverse primer, 2.5ul of Taq buffer (1X) and 5 Units of Taq DNA polymerase. Thermal cycling includes preliminary denaturation of the gDNA at 95°C for 5 minutes, followed by 35 rounds of denaturation at 95°C for 30 seconds, annealing of primer at 56.5°C for 30 seconds, extension at 72°C for 45 seconds, and a final extension step at 72°C for 7 minutes. Products of PCR were kept at incubation in a water bath for 14–16 hours at 37°C with Psp5II (PpuMI) restriction enzyme (Catalog # ER0761, Thermofisher Scientific). Psp5II generates fragments of 141bp and 224bp in presence of the dominant C allele, 365bp fragment is left uncut for the recessive T allele as presented in Fig 1. To visualize the restriction products in a gel electrophoresis system, two percent agarose (ultraPure^™^, Carlsbad, CA. 92008) gel was prepared. Gel images were taken and kept in a gel documentation system (Basel, Switzerland).

### Measurement of pro-inflammatory cytokines

Pro-inflammatory cytokines in plasma were measured according to the directions provided in the Kit (TNF-α;kit ab181421, IL-1β;ab46052, and IL-6 ab178013) (The Eagle Biosciences) using a spectrophotometer (Kenza spectrophotometer (Number; 450 TX), Biolabo, France. This assay is based on the quantitative sandwich enzyme immunoassay technique. A monoclonal antibody specific for pro-inflammatory cytokine was pre-coated onto a microplate. Samples were pipetted into the wells and the specific cytokine bound by the immobilized antibody. Unbound samples were removed by washing and a detection antibody specific for the particular cytokine was added to the wells. Then it attach to the capture antibody-cytokine in sample. Washing step was performed again and enzyme conjugate was added to the wells then substrate was added following incubation and washing. A coloured product was formed in proportion to the amount of pro-inflammatory cytokine present in the sample. Then the reaction was terminated by adding an acid and absorbance was measured at 450nm.

### Statistical analysis

Mann Whitney test using SPSS software of version 20.0 (IBM Corp., Armonk, NY, USA) was applied to find the statistical significance. α ≤ 0.05 was set for statistical significance. Chi-square and Fisher exact test were used to analyze genotypes and the frequency of alleles. Odd ratio (OR) with 95% Confidence Interval (CI). Genotype data were analysed by GraphPad Prism version 7.0 software (CA, USA).

## Results

### Semen quality

Several sperm parameters statistics like concentration, volume, and pH have been shown in Table 2. Sperm concentration for azoospermia males showed none of the sperms while for oligospermia and oligoasthenospermia (30.22 and 23.99 million/mL) it was substantially less than the non-infertile (fertile) group (138.39 million/mL). Only the asthenospermic group (72.82 million/mL) showed more sperm concentration than other infertility groups but still it is less than the fertile group. Mean pH value for all the infertility groups was found to be slightly higher than the non-infertile group (7.35). The mean volume of semen samples was greater in non-infertile group (3.73mL) than all of the infertile groups.

**Table 2:** Statistics of semen parameters for Infertile and non-infertile subjects.

Groups (N)	Parameters	Sperm concentration (million/mL)	pH	Age (Years)	Liquefaction time (Minutes)	Period of sexual absence (Days)	Volume (mL)
**Oligospermia (12)**	Mean ± S. D	30.22* ± 4.04	7.42 ± 0.05	30.83 ± 1.84	31.25 ± 1.75	6.25 ± 0.57	3.12 ± 0.24
**Oligoasthenospermia (21)**	Mean ± S. D	23.99* ± 3.65	7.47 ± 0.05	34.05 ± 1.66	28.33 ± 1.05	6.38 ± 0.51	3.39 ± 0.28
**Azoospermia (7)**	Mean ± S. D	_	7.5 ± 0.06	37.86 ± 1.87	30.71 ± 2.02	12.14 ± 2.66	3.64 ± 0.56
**Asthenospermia (5)**	Mean ± S. D	72.82* ± 11.62	7.44 ± 0.07	36.40 ± 1.72	31.00 ± 2.45	7.20 ± 0.86	3.50 ± 0.27
**Non-infertility (20)**	Mean ± S. D	138.39 ± 15.41	7.35 ± 0.03	31.25 ± 1.46	33.00 ± 1.05	5.70 ± 0.35	3.73 ± 0.19

*statistically significant at p<0.05

### RBC-AChE in infertile and non-infertile groups

**Fig 2:**
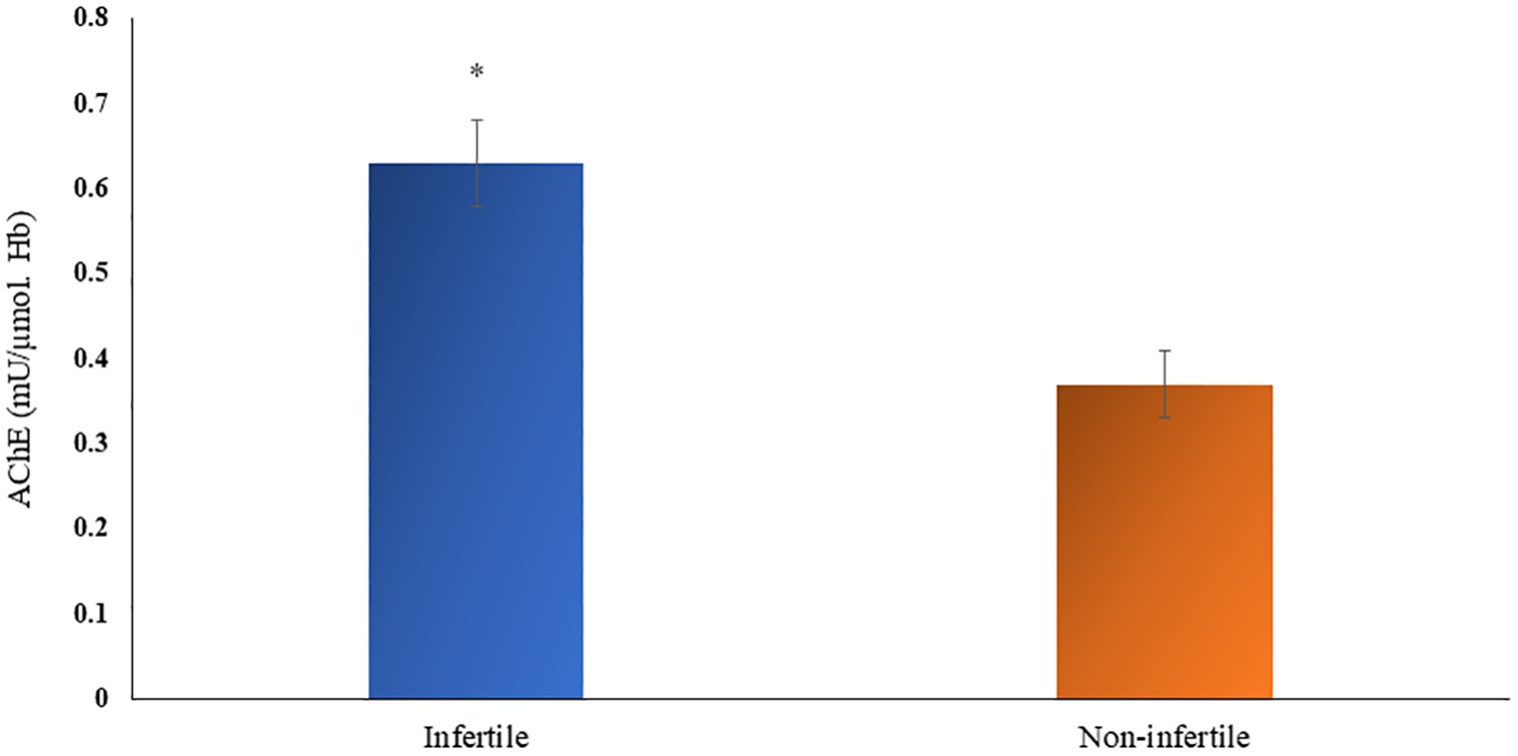
Activity of acetylcholinesterase in infertile and non-infertile groups, (* significant at p<0.05).

Acetylcholinesterase activity in non-infertile and infertile males is depicted in Fig 2. A significantly higher level (p≤0.001; independent sample t-test) of AChE enzyme activity was found in the infertile group (0.64±0.26 mU/μmol Hb, 95% CI 0.56–0.72; n = 42) as compared to non-infertile cohort (0.29±0.28 mU/μmol Hb, 95% CI 0.21–0.36; n = 50).

### Pro-inflammatory cytokines in infertile and non-infertile groups

Table 3 illustrates the status of pro-inflammatory cytokines in plasma samples of non-infertile and infertile males. Statistically increased IL-1β level (4.68 ng/L± 0.26) was noted in infertile males as compared to non-infertile males (3.24 ng/L ±0.24). IL-1β was also shown to be statistically significant (p≤ 0.01) for oligoasthenospermia, oligospermia and asthenospermia. IL-6 and TNF-α were higher in the infertile group but they were not statistically significant. Tables 4–6 shows the status of IL-1β, IL-6, and TNF-α in different types of male infertility. The mean values of IL-1β for all kinds of infertility are greater than the non-infertile group (3.24ng/L). For IL-6 and TNF- α, only the asthenospermic group showed less mean value (2.99 and 2.46ng/L respectively) than the non-infertile group. Out of all the three cytokines measured, primary and secondary infertility showed statistical significance (p≤ 0.01) only for IL-1β.

**Table 3:** Status of Pro-inflammatory cytokines in infertile and non-infertile cohorts.

Pro-inflammatory Cytokines	Group	N	Mean (ng/L)	SE	95% [CI]	p-value (Significant at p≤0.05)
**IL-1β**	Infertile	45	4.68	0.26	4.14–5.21	0.000
	Non-infertile	42	3.24	0.24	2.74–3.74	_
**IL-6**	Infertile	45	4.05	0.38	3.27–4.83	0.228
	Non-infertile	42	3.59	0.28	3.02–4.15	_
**TNF-α**	Infertile	45	3.33	0.36	2.60–4.07	0.281
	Non-infertile	42	2.69	0.30	2.08–3.31	_

**Table 4:** Status of IL-1β indifferent types of male infertility.

Groups	N	Mean (ng/L)	S. E	95% [C.I]	p-value
**Primary infertility**	37	4.78	0.32	4.14–5.43	0.000
**Secondary infertility**	8	4.19	0.27	3.56–4.83	0.004
**Oligospermia**	12	4.68	0.27	4.07–5.30	0.000*
**Oligoasthenospermia**	21	4.83	0.44	3.91–5.75	0.000
**Azoospermia**	7	4.58	1.05	2.00–7.16	0.063
**Asthenospermia**	5	4.26	0.35	3.29–5.23	0.010
**Non-infertility**	42	3.24	0.25	2.74–3.74	_

**Table 5:** Status of IL-6 in different types of male infertility.

Groups	N	Mean (ng/L)	S. E	95% [C.I]	p-value
**Primary infertility**	37	4.30	0.46	3.37–5.23	0.133
**Secondary infertility**	8	2.90	0.18	2.47–3.33	0.771
**Oligospermia**	12	4.27	0.65	2.81–5.73	0.164
**Oligoasthenospermia**	21	4.36	0.66	2.98–5.74	0.166
**Azoospermia**	7	3.65	1.14	0.86–6.44	0.520
**Asthenospermia**	5	2.99	0.21	2.41–3.56	0.945
**Non-infertility**	42	3.59	0.28	3.02–4.16	_

**Table 6:** Status of TNF-α in different types of male infertility.

Groups	N	Mean (ng/L)	S. E	95% [C.I]	p-value
**Primary infertility**	37	3.44	0.41	2.60–4.28	0.242
**Secondary infertility**	8	2.86	0.77	1.03–4.69	0.832
**Oligospermia**	12	3.75	0.95	1.64–5.86	0.380
**Oligoasthenospermia**	21	3.25	0.51	2.17–4.32	0.493
**Azoospermia**	7	3.24	0.91	1.01–5.48	0.511
**Asthenospermia**	5	2.46	0.57	0.88–4.05	0.972
**Non-infertility**	42	2.69	0.30	2.08–3.31	_

### Genotype distribution and alleles frequency in *ACHE* gene SNP rs 17228602

**Fig 3:**
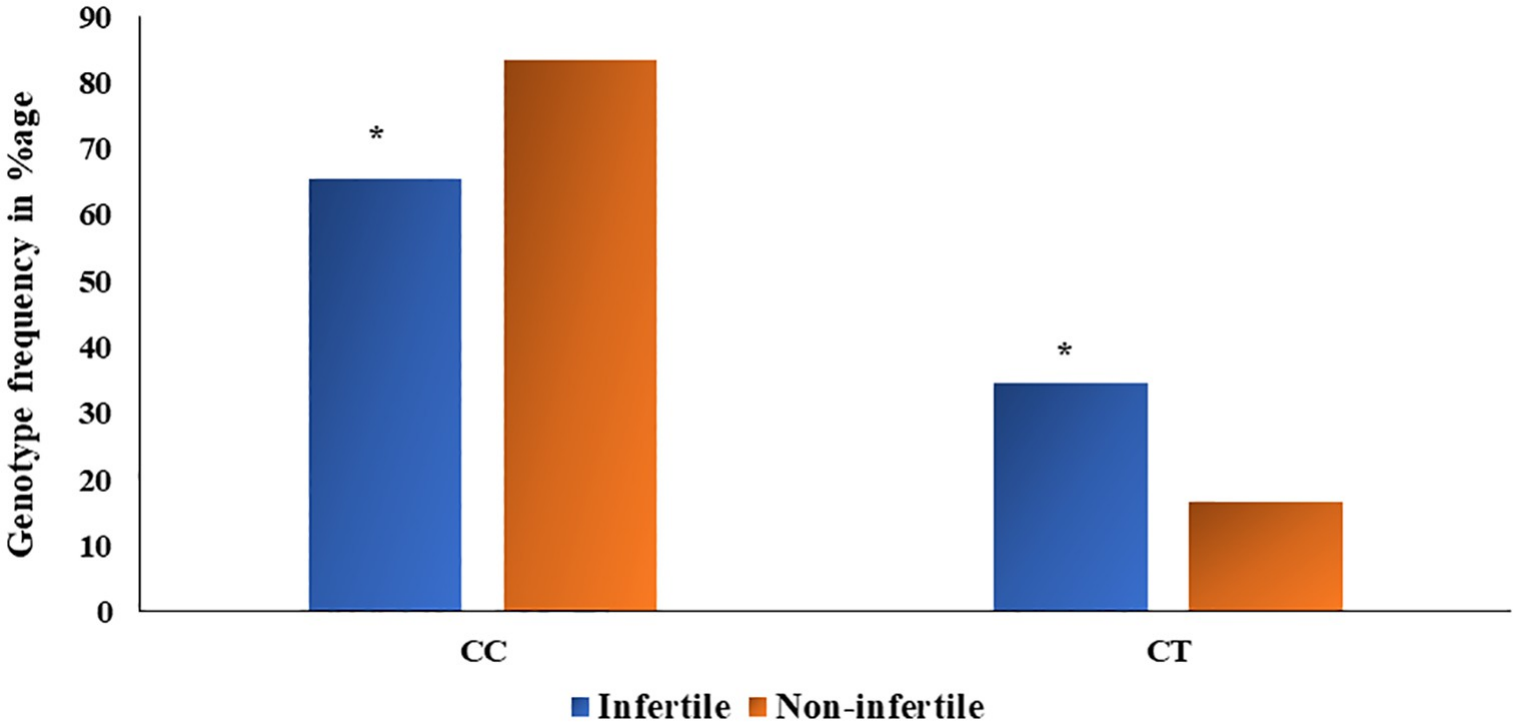
Genotype frequency distribution in percentage (CC and CT) of rs17228602 *AChE* SNP in infertile and non-infertile male group. (* significant at p<0.05).

**Fig 4:**
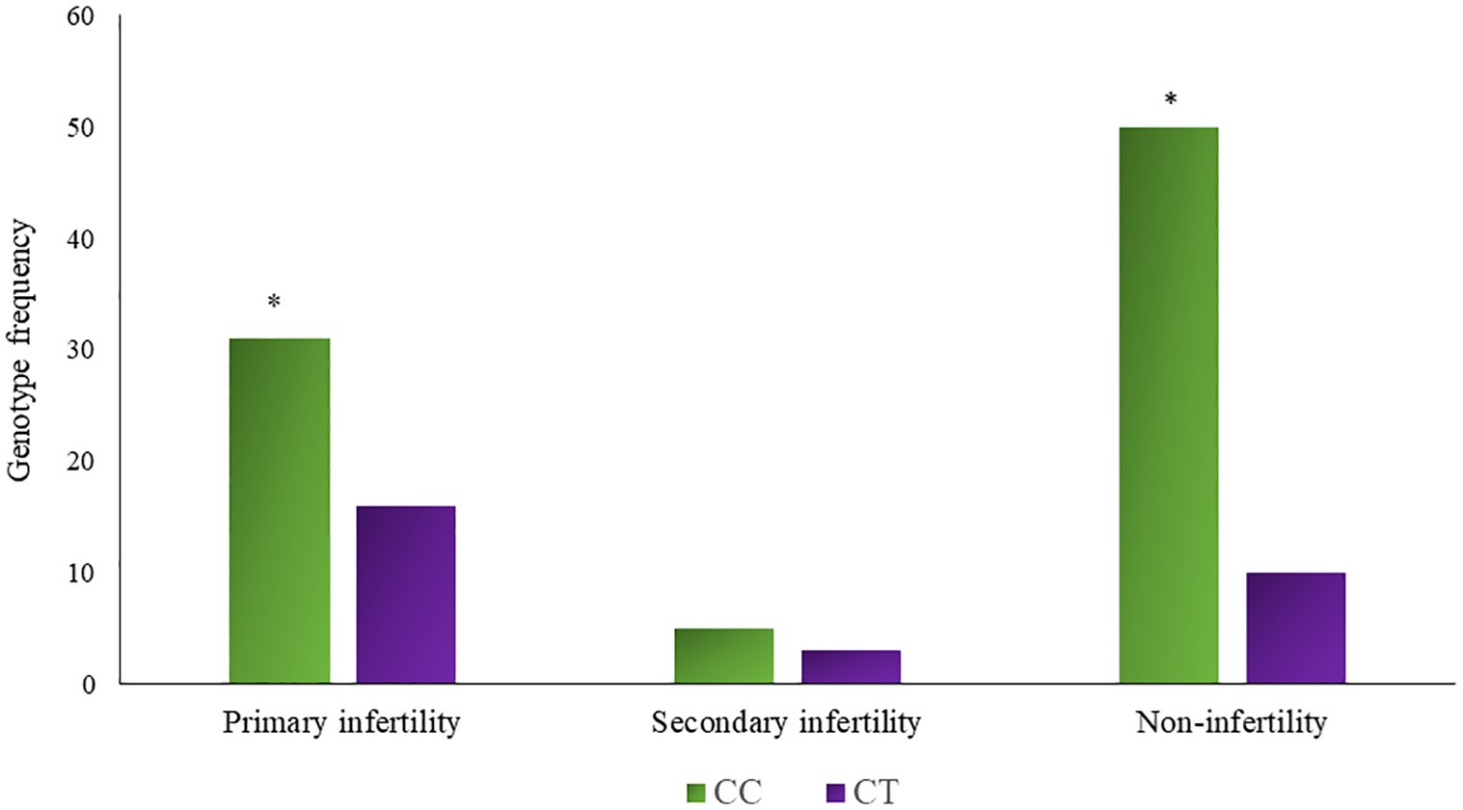
Genotype frequency (CC and CT) of rs17228602 *ACHE* SNP in Primary and Secondary infertility. * Shows the significance at p<0.05.

**Fig 5:**
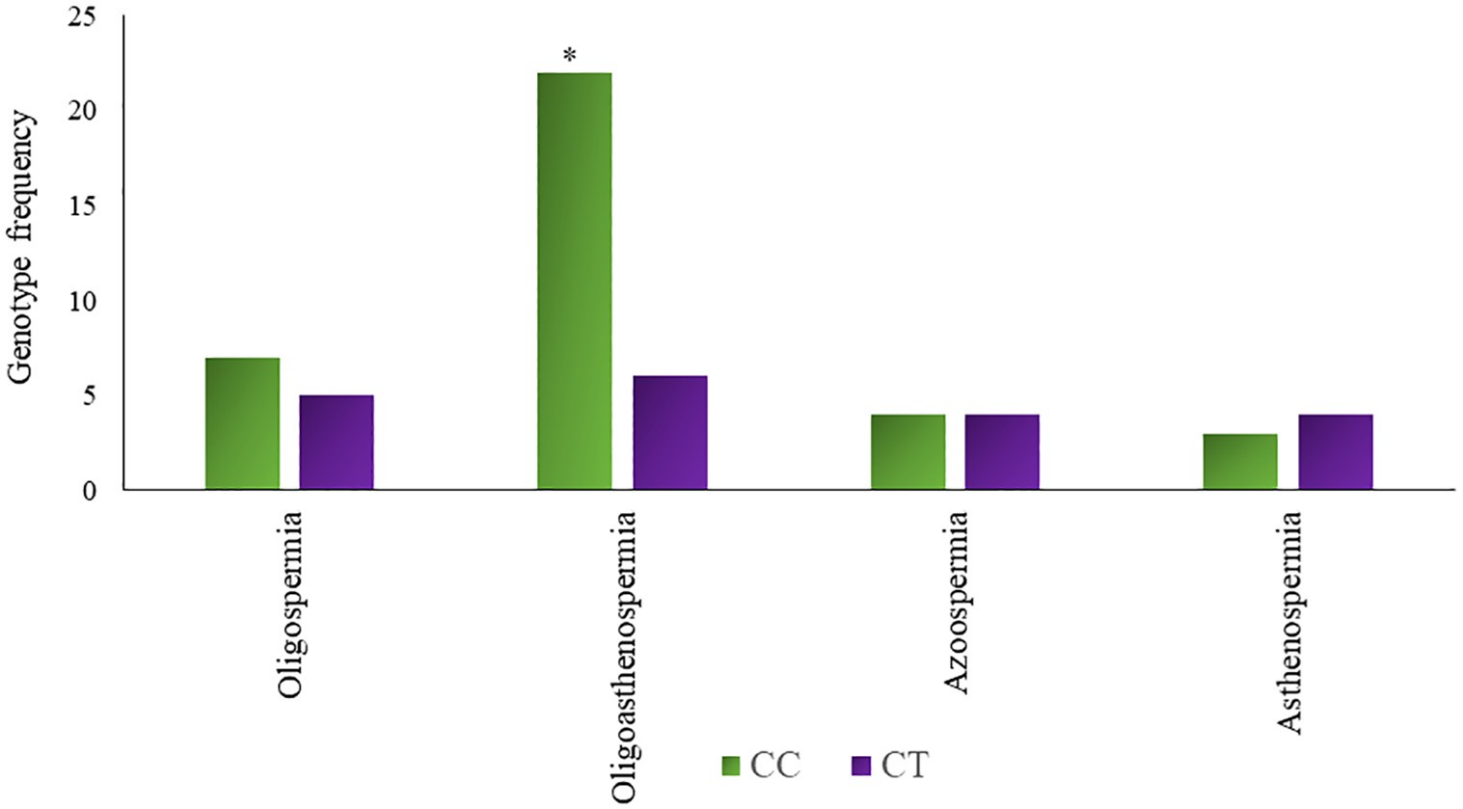
Genotype frequency (CC and CT) of rs17228602 *AChE* SNP in different types of infertility. * Shows the significance at p<0.05.

Hardy Weinberg Equilibrium (HWE) was determined for the genotype distribution of the understudied non-infertile and infertile males. Divergence from HWE in infertile and fertile groups was noted (χ2 = 2.397, P = 0.121; χ2 = 0.496, P = 0.481), most probably due to the absence of homozygous minor allele TT genotype and small sample size. However, it was not statistically significant. Genotypes and allelic frequencies of rs 17228602 (C>T) in non-infertile and infertile males are presented in Table 7. Noticeable variance in genotype distribution between the non-infertile and the infertile male group was found (χ2 = 3.962, p = 0.04) as shown in Fig 3. Frequency of CT genotype was significantly high in infertile males (34.54%) as compared to non-infertile males (16.66%); CC genotype frequency came out to be 65.45% while no TT genotype was present in both male groups. Moreover, significant association of rs17228602 minor allele genotype in dominant genetic model was noted (OR = 0.378, 95% CI = 0.157–0.911, p = 0.04). Major C allele frequency was 82.72% in infertile and 91.66% in non-infertile men while minor T allele frequency was increased in the infertile group (17.27%) than in non-infertile (8.33%). Figs 4 and 5 depict the types of prevalent infertility concerning genotypes.

**Table 7:** Genotypes and allelic frequency in *AChE* gene SNP rs 17228602 in non-infertile and infertile males.

Genotype and allelic frequencies	Infertile men, N (%)	Non-infertile men, N (%)	Odd ratio (95% C.I)	Chi-square (*χ2*)	p-value
**Genotype**					
CC	36 (65.45%)	50 (83.33%)		3.962	0.04
CT	19 (34.54%)	10 (16.66%)			
TT	0 (0%)	0 (0%)			
DM = CC vs CT +TT			0.378 (0.157–0.911)	3.962	0.04
**Allele**					
C	91 (82.72%)	110 (91.66%)	0.435 (0.192–0.983)	3.390	0.06
T	19 (17.27%)	10 (8.33%)			

## Discussion

The pathophysiology of male infertility is multifaceted. Studies have shown the role of cholinergic enzymes in the etiology of male infertility [41]. The role of the cholinergic system in male sexual health has been reviewed and described by [42] in the handbook of clinical neurology In the present study, an increase in blood AChE level and pro-inflammatory cytokines; IL-1β, IL-6, and TNF-α were observed, suggesting the involvement of cholinergic system and low-grade systemic inflammation in infertility condition. In addition, an association of *ACHE* gene SNP rs17228602 was evident with the infertile males. It is noteworthy that type 2 diabetes, obesity, and insulin resistance are proven low-grade systemic inflammatory conditions that impair reproductive functioning.

Acetylcholine produces an inflammatory response through the vagus nerve in the peripheral nervous system [43]. The circulating ACh in plasma/serum or tissue may decrease due to increased AChE, resulting in dysregulation of the immune system [41]. On the other hand, increased AChE has been reported in many low-grade inflammatory diseases like Alzheimer’s and type 2 diabetes [44, 45]. In Alzheimer, AChE inhibitors are the first line of drug for treatment. The presence of AChE in seminal fluid, human prostrate, and sperm is documented in the literature [46]. The activity of AChE turned out to be decreased in samples with atypical seminal parameters like sperm concentration and motility [46]. According to the literature, acetylcholinesterase read-through variant (AChE-R) activity is overexpressed in stress-induced male infertility [30] whereas AChE-R is depicted to govern by miR125b, one of the microRNAs regulated by *ACHE* SNPs present in 3 UTR region [28]. Based on this fact, it may be assumed that the faulty communication of miR-125b/ACHE variant rs17228602 adversely affects the relevant regulation of cholinergic signalling and pathways, leading to pathophysiological conditions like reproductive infertility. The impact of the 3’ UTR sequence in ACHE signalling is obvious by the very fact that knock-out mice of this sequence show cognitive decline due to stress [47]. Psychological stress is associated with reduced male fertility involving glucocorticoid components. Glucocorticoid function is to prevent inflammation and its association with the AChE-R variant is a sign of its involvement in the pathology of male infertility. The elevated AChE activity in infertile males as observed in the present study is suggestive of low-grade systemic inflammation [41]. Dysregulated AChE has been reported in many low-grade systemic inflammatory diseases [48] and a causal relationship between inflammatory pathways and cholinergic signalling [49, 50]. Higher activity of AChE has been linked with several other pathological conditions in earlier research [48, 51–53]. Mechanistically, the rise in the AChE means less availability of ACh for physiological roles because of the rapid hydrolysis of ACh. Consequently, the cholinergic anti-inflammatory pathway which is mediated by vagus nerve cells in peripheral nervous systems is presumably dysregulated. It is worthwhile to mention that direct or indirect evidence for the innervation of the vagus nerve to the male genitalia has been shown in the literature [54, 55].

Pro-inflammatory cytokines are proteins, glycoproteins, or peptides that function as signalling molecules and are secreted by immune cells. Pro-inflammatory cytokines promote inflammation by targeting cells. It is clear from the literature that the first pro-inflammatory cytokine was released by the somatic cells of the male reproductive tract. Changes in immune homeostasis that are associated with inflammation can cause male infertility [6]. Moreover, the rise in cytokine levels directly or indirectly affects sperm motility [56]. The influence of considerable amounts of cytokines may harm the sperm’s DNA level and integrity. These findings imply that an increase in pro-inflammatory cytokines is a foremost risk factor to develop male sterility. TNF-α was found to be increased in infertile males in the present study. TNF-α regulates spermatogenesis and has been reported to be increased unexplained infertility [57]. IL-6 level increases in dyspermia cases and shows a negative correlation with spermiogram parameters [58]. In addition, an increase in pro-inflammatory cytokines in the blood can disrupt the blood-testis barrier in the body. Blood-testis barrier is also affected by Interleukin-6 by delaying the degradation of some proteins [34]. Inflammation directly affects sperm production during spermatogenesis affecting a male’s fecundity [59]. Inflammation is also linked with elevated levels of reactive oxygen species. Increased reactive oxygen species and dysregulated antioxidant enzymes have been reported to cause male infertility [15, 36, 53]. Molecular analysis of *ACHE* gene SNP rs17228602 revealed a significant (p = 0.000) association with male infertility (Table 7). A significant association of the rs17228602 variant genotypes CT with the risk to develop male infertility in analyzed samples was observed (Fig 3). Javed *et al*., [28] found an association of this variant with the vulnerability of addiction and increased AChE was mentioned in different addicted groups. However, Furqan et al. [57] did not find any association of this SNP with cannabis addiction. The study on the association of the rs17228602 variant with vulnerability to male infertility has not been reported earlier. The SNP is present in the 3’UTR region. Polymorphism in this region can transcriptionally affect many genes by regulating mRNA and micro-RNA [29].

Further studies on the subject may help in a better understanding of idiopathic and unexplained male infertility. In addition, the development of new therapeutic approaches and diagnostic markers based on cholinergic enzymes, pro-inflammatory cytokines, or AChE-regulating miRNA may be proposed.

## Conclusion

AChE enzyme and *ACHE gene SNP* rs17228602 are involved in male infertility, especially in primary infertility, oligospermia, and oligoasthenospermia. Pro-inflammatory cytokine IL-1β was significantly dysregulated while TNF-α and IL-6 were noticeably increased in infertile males, considering the possibility of cholinergic-mediated systemic inflammation in male infertility. However, further study with larger sample size and different *ACHE* SNPs is suggested. In addition, research on cholinergic-anti-inflammatory pathways and AChE-regulating miRNA may provide a better understanding of idiopathic and unexplained cases of male infertility.

## Supporting information

S1 Fig Original image. (TIFF)

S1 Table Dataset. (PDF)

S2 Table Fertile rflp data. (PDF)

S3 Table Infertile rflp data. (PDF)
