# Supplementary figures and images for: Acetylcholinesterase, pro-inflammatory cytokines, and association of ACHE SNP rs 17228602 with male infertility
Source: PLoS One. 2023 Apr 7;18(4):e0282579. doi: 10.1371/journal.pone.0282579 (PMC10081795; doi:10.1371/journal.pone.0282579)

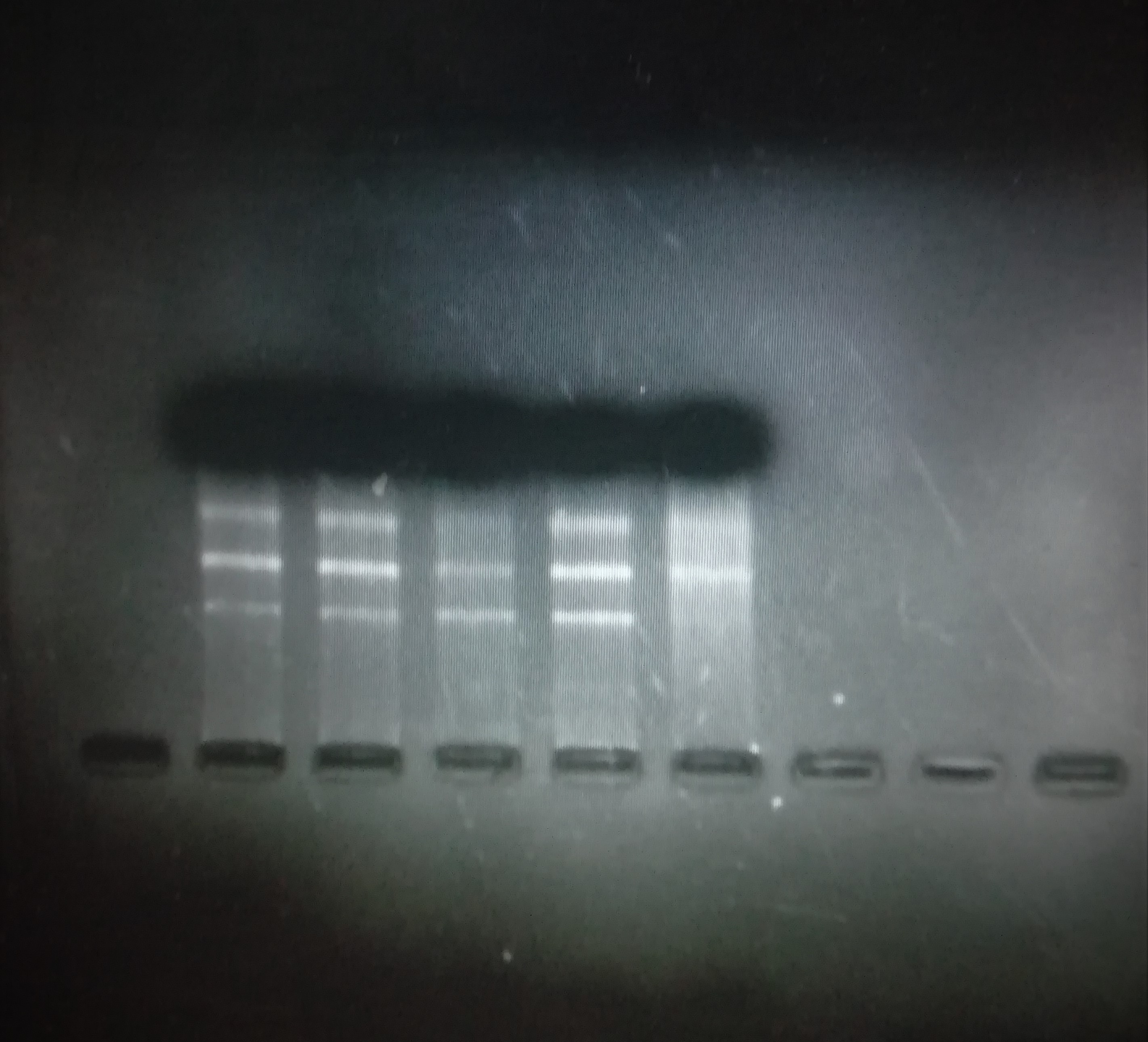

Supplement: S1 Fig — (TIFF) [file pone.0282579.s001.tiff]
