# Supplementary material for: Acetylcholinesterase, pro-inflammatory cytokines, and association of ACHE SNP rs 17228602 with male infertility
Source: PLoS One. 2023 Apr 7;18(4):e0282579. doi: 10.1371/journal.pone.0282579 (PMC10081795; doi:10.1371/journal.pone.0282579)
Supplement: S1 Table — (PDF) [file pone.0282579.s002.pdf]

Sample ID	IL1-B	IL-6	TNF	Type of Infertility	Sperm Concentration Million/ml
M1	5.19	4.43	5.35	Primary	54.67
M2	4.36	6.14	5.9	Primary	8.5
M3	2.78	2.11	2.29	Primary	0
M4	3.49	2.42	3.3	Primary	0
M5	3.83	2.41	0.85	Secondary	90.88
M6	4.02	3.07	0.91	Primary	46.24
M7	5	4.54	6.41	Primary	54.74
M8	4.26	2.32	1.32	Primary	0
M9	5.36	8.77	3.65	Primary	32.95
M10	3.54	2.57	5.05	Secondary	45.18
M11	3.14	2.47	2.65	Primary	4.25
M12	4.13	2.71	1.15	Secondary	46.77
M13	3.77	2.7	3.58	Primary	99.39
M14	4.84	3.54	2.62	Primary	3.19
M15	4.53	3.4	1.05	Primary	10
M16	5.94	3.91	1.83	Secondary	25
M17	5.56	5.61	1.85	Primary	5
M18	4.59	2.65	1.65	Primary	40
M19	10.75	10.45	7.63	Primary	0
M20	4.09	2.99	2.55	Primary	80
M21	2.79	2.47	5.21	Primary	0
M22	3.94	2.76	0.81	Primary	40.74
M23	3.85	2.88	5.36	Primary	50.23
M24	6.27	7.61	4.12	Primary	15.6
M25	3.75	2.65	2.21	Secondary	0
M26	3.73	2.99	3.56	Secondary	30.88
M27	4.27	3.45	5.73	Primary	300
M28	4.45	4.37	0.94	Primary	40.74
M29	3.72	2.54	0.59	Primary	15.5
M30	5.48	3.56	1.31	Primary	19
M31	12.77	16.09	7.39	Primary	25.3
M32	3.51	2.19	3.21	Primary	10
M33	5.27	5.32	1.56	Primary	23
M34	5	5.14	11.44	Primary	17.43
M35	4.24	3.11	0.76	Primary	0
M36	5.64	3.61	1.53	Primary	35.2
M37	3.82	3.37	0.78	Primary	8.9
M38	4.58	3.28	2.36	Primary	12
M39	2.48	2.16	4.9	Primary	7
M40	4.27	2.55	7.01	Secondary	24.45
M41	3.97	3.23	3.8	Primary	58.65
M42	6.52	7.86	4.37	Primary	18.7
M43	4.65	2.93	7.39	Primary	25
M44	4.11	3.66	1.07	Primary	45.5
M45	4.35	3.4	1.24	Secondary	30

Motility	Immotility (%)	Infertility
24%	73%	Oligoasthenospermia
1%	99%	Oligoasthenospermia
0%	0%	Azoospermia
0%	0%	Azoospermia
32%	59%	Asthenospermia
49%	51%	Oligospermia
60%	40%	Oligospermia
0%	0%	Azoospermia
46%	50%	Oligospermia
48%	52%	Oligospermia
22%	78%	Oligoasthenospermia
38%	57%	Oligoasthenospermia
13%	78%	Asthenospermia
6%	84%	Oligoasthenospermia
10%	75%	Oligoasthenospermia
20%	70%	Oligoasthenospermia
25%	60%	Oligoasthenospermia
45%	55%	Oligospermia
0%	0%	Azoospermia
36%	64%	Asthenospermia
0%	0%	Azoospermia
45%	55%	Oligoasthenospermia
25%	75%	Oligoasthenospermia
2%	98%	Oligoasthenospermia
0%	0%	Azoospermia
51%	49%	Oligospermia
99%	1%	Oligospermia
40%	60%	Oligoasthenospermia
55%	45%	Oligospermia
60%	40%	Oligospermia
40%	60%	Oligoasthenospermia
30%	70%	Oligoasthenospermia
25%	75%	Oligoasthenospermia
70%	30%	Oligospermia
0%	0%	Azoospermia
35%	75%	Asthenospermia
40%	60%	Oligoasthenospermia
57%	43%	Oligospermia
20%	80%	Oligoasthenospermia
30%	70%	Oligoasthenospermia
35%	65%	Asthenospermia
60%	40%	Oligospermia
40%	60%	Oligoasthenospermia
45%	55%	Oligoasthenospermia
25%	75%	Oligoasthenospermia
