# Supplementary material for: Acetylcholinesterase, pro-inflammatory cytokines, and association of ACHE SNP rs 17228602 with male infertility
Source: PLoS One. 2023 Apr 7;18(4):e0282579. doi: 10.1371/journal.pone.0282579 (PMC10081795; doi:10.1371/journal.pone.0282579)
Supplement: S2 Table — (PDF) [file pone.0282579.s003.pdf]

sample	CC	CT	TT	C Allele	T Allele
CT1	1	0	0	2	0
CT2	1	0	0	2	0
CT3	1	0	0	2	0
CT4	1	0	0	2	0
CT5	1	0	0	2	0
CT6	1	0	0	2	0
CT7	1	0	0	2	0
CT8	1	0	0	2	0
CT9	1	0	0	2	0
CT10	1	0	0	2	0
CT11	1	0	0	2	0
CT12	1	0	0	2	0
CT13	1	0	0	2	0
CT14	1	0	0	2	0
CT15	1	0	0	2	0
CT16	0	1	0	1	1
CT17	1	0	0	2	0
CT18	1	0	0	2	0
CT19	1	0	0	2	0
CT20	1	0	0	2	0
CT21	1	0	0	2	0
CT22	0	1	0	1	1
CT23	1	0	0	2	0
CT24	1	0	0	2	0
CT25	1	0	0	2	0
CT26	1	0	0	2	0
CT27	1	0	0	2	0
CT28	1	0	0	2	0
CT29	0	1	0	1	1
CT30	1	0	0	2	0
CT31	1	0	0	2	0
CT32	0	1	0	1	1
CT33	1	0	0	2	0
CT34	0	1	0	1	1
CT35	1	0	0	2	0
CT36	1	0	0	2	0
CT37	1	0	0	2	0
CT38	1	0	0	2	0
CT39	1	0	0	2	0
CT40	1	0	0	2	0
CT41	0	1	0	1	1
CT42	1	0	0	2	0
CT43	1	0	0	2	0
CT44	1	0	0	2	0
CT45	0	1	0	1	1
CT46	0	1	0	1	1
CT47	0	1	0	1	1
CT48	1	0	0	2	0
CT49	1	0	0	2	0

CT50	1	0	0	2	0
CT51	1	0	0	2	0
CT52	1	0	0	2	0
CT53	1	0	0	2	0
CT54	1	0	0	2	0
CT55	1	0	0	2	0
CT56	1	0	0	2	0
CT57	1	0	0	2	0
CT58	1	0	0	2	0
CT59	0	1	0	1	1
CT60	1	0	0	2	0
	50	10	0	110	10
