# Supplementary material for: Acetylcholinesterase, pro-inflammatory cytokines, and association of ACHE SNP rs 17228602 with male infertility
Source: PLoS One. 2023 Apr 7;18(4):e0282579. doi: 10.1371/journal.pone.0282579 (PMC10081795; doi:10.1371/journal.pone.0282579)
Supplement: S3 Table — (PDF) [file pone.0282579.s004.pdf]

Sample	CC	CT	TT	C Allele	T Allele
1	0	1	0	1	1
2	0	1	0	1	1
3	0	1	0	1	1
4	0	1	0	1	1
5	1	0	0	2	0
6	1	0	0	2	0
7	0	1	0	1	1
8	1	0	0	2	0
9	1	0	0	2	0
10	0	1	0	1	1
11	0	1	0	1	1
12	0	1	0	1	1
13	1	0	0	2	0
14	1	0	0	2	0
15	1	0	0	2	0
16	1	0	0	2	0
17	1	0	0	2	0
18	1	0	0	2	0
19	1	0	0	2	0
20	0	1	0	1	1
21	1	0	0	2	0
22	1	0	0	2	0
23	0	1	0	1	1
24	1	0	0	2	0
25	0	1	0	1	1
26	0	1	0	1	1
27	1	0	0	2	0
28	1	0	0	2	0
29	1	0	0	2	0
30	1	0	0	2	0
31	1	0	0	2	0
32	1	0	0	2	0
33	1	0	0	2	0
34	1	0	0	2	0
35	0	1	0	1	1
36	0	1	0	1	1
37	0	1	0	1	1
38	0	1	0	1	1
39	1	0	0	2	0
40	1	0	0	2	0
41	1	0	0	2	0
42	1	0	0	2	0
43	1	0	0	2	0
44	1	0	0	2	0
45	1	0	0	2	0
46	1	0	0	2	0
47	1	0	0	2	0
48	1	0	0	2	0
49	1	0	0	2	0

50	1	0	0	2	0
51	0	1	0	1	1
52	1	0	0	2	0
53	1	0	0	2	0
54	0	1	0	1	1
55	0	1	0	1	1
	36	19	0	91	19
